# Treatment of gram - positive infections in critically ill patients

**DOI:** 10.1186/1471-2334-14-92

**Published:** 2014-11-28

**Authors:** Cristina Vazquez-Guillamet, Marin H Kollef

**Affiliations:** Division of Infectious Diseases, University of New Mexico, Albuquerque, Mexico; Division of Pulmonary and Critical Care Medicine, Washington University School of Medicine, St. Louis, Missouri

**Keywords:** Gram-positive cocci, Antibiotics, *Staphylococcus aureus*, Enterococci, Resistance

## Abstract

Gram-positive bacteria to include methicillin-resistant *Staphylococcus aureus* (MRSA), methicillin-susceptible *Staphylococcus aureus* (MSSA), and enterococci, to include vancomycin-resistant enterococci (VRE), display a remarkable array of resistance and virulence factors, which have contributed to their prominent role in infections of the critically ill. Over the last three decades infections with these pathogens has increased as has their overall resistance to available antimicrobial agents. This has led to the development of a number of new antibiotics for the treatment of Gram-positive bacteria. At present, it is important that clinicians recognize the changing resistance patterns and epidemiology of Gram-positive bacteria as these factors may impact patient outcomes. The increasing range of these pathogens, such as the emergence of community-associated MRSA clones, emphasizes that all specialties of physicians treating infections should have a good understanding of the infections caused by Gram-positive bacteria in their area of practice. When initiating empiric antibiotics, it is of vital importance that this therapy be timely and appropriate, as delays in treatment are associated with adverse outcomes. Although vancomycin has traditionally been considered a first-line therapy for serious MRSA infections, multiple concerns with this agent have opened the door for alternative agents demonstrating efficacy in this role. Similarly, the expansion of VRE as a pathogen in the ICU setting has required the development of agents targeting this important pathogen.

## Scope of the problem

Sepsis represents a major heath care problem with half of the cases occurring in the critically ill and it is associated with a high mortality (50% for septic shock) for intensive care unit (ICU) patients [1, 2]. The administration of early appropriate antibiotics is recognized as one of the most important interventions linked to improving patient outcomes in sepsis [3–5]. The microbiology in the ICU has changed in the last 2 to 3 decades so that Gram-positive cocci (GPC) now represent one of the dominant species. A recent survey showed that GPC cause the majority of nosocomial infections with *Staphylococcus aureus* (16%, with more than 50% being methicillin-resistant [MRSA]) and *Enterococcus* species (14%, with vancomycin-resistant enterococci [VRE] accounting for approximately 3.5% of all infections) predominating [6]. New resistance patterns are also emerging to include vancomycin - intermediate *Staphylococcus aureus* (VISA), increases in the *Staphylococcus aureus* minimum inhibitory concentration (MIC) to vancomycin without breaching the resistance threshold (i.e., MIC creep), vancomycin-resistant *Staphylococcus aureus* (VRSA) due to acquisition of the vanA gene, as well as daptomycin and linezolid resistance. Given these newly described resistance patterns, testing for susceptibility and adequate antibiotic dosing are of paramount importance for proper management of critically ill infected patients.

For the purpose of this review we will focus on the contribution of GPC to infections in critically ill patients emphasizing the agents available for their treatment. In the ICU, respiratory tract infections especially pneumonia, represent the most common infection and carry the highest mortality [2]. The microbiology of pneumonia varies considerably based on the presence of risk factors for antibiotic resistance. While most community-acquired pneumonia (CAP) cases are caused by *Streptococcus pneumoniae*, health care associated pneumonias (HCAPs), particularly ventilator-associated pneumonia (VAP), are often caused by MRSA. Community-acquired MRSA pneumonia can also occur and accounts for 3% of bacterial pneumonia cases [7], usually being associated with younger patients, post-influenza, and necrotizing pneumonia. The rates of penicillin and ceftriaxone resistant strains of *Streptococcus pneumoniae* are relatively low in adults [8]. However, macrolide resistance can be seen in up to 30% of strains. Risk factors for resistant pathogens appear to be identical for both CAP and HCAP and include: prior hospitalization and antibiotics, immunosuppression, non-ambulatory status, tube feeds and gastric acid suppressive agents [9].

With the advance of invasive devices (e.g. ventricular assisted devices, intravenous catheters) has come a rise in the incidence of bacteremia due to GPC. Along with device removal and a meticulous search for metastatic foci of infection (discitis, osteomyelitis, epidural abscess), antibiotic treatment remains the cornerstone of therapy. As will be discussed various choices are available for the treatment of bacteremia due to GPC. When *Staphylococcus aureus* is suspected, combination therapy with an anti-staphylocccal penicillin (nafcillin, oxacillin) and vancomycin should be considered until susceptibility results are known [10]. Daptomycin has emerged as a good alternative agent for *Staphylococcus aureus* bacteremia and endocarditis [11]. It also offers the advantage of proven efficacy in patients with MRSA bacteremia with vancomycin MIC >1 mg/L and for infections attributed to heteroresistant VISA, but not for VRSA [12, 13]. Linezolid has also been shown to have good activity as compared to vancomcyin in *Staphylococcus aureus* bacteremia [14].

Although less common than pneumonia and bacteremia, complicated skin and soft tissue infections (SSTIs) can be grave enough to warrant ICU care. Also, postsurgical site infections can complicate ICU stays. The main pathogen isolated in these infections is MRSA which makes empirical coverage mandatory [15]. In recent years, most new drugs targeting GPC (e.g. linezolid, ceftaroline, telavancin, daptomcyin, tigecycline) have come to market by gaining indication for treatment of SSTIs. Moreover, there are now recognized subpopulations of patients with SSTIs who are at increased risk of bacteremia necessitating more aggressive and prolonged therapy [16, 17].

Usually dominated by Gram - negative rods and anaerobes, health-care associated intra-abdominal infections in debilitated patients often require empirical coverage for enterococci including VRE. The true pathogenicity of enterococci in these polymicrobial infections remains unclear, but isolation of enterococci from peritoneal fluid in severe infections was found to be an independent predictor of mortality [18]. So far, limited data are available to formulate guideline recommendations for the coverage of GPC except for VRE coverage in certain high-risk patient populations (liver transplant recipients, post-surgical complications in patients with prior antibiotics, patients undergoing hepatobilliary surgery, patients with known VRE colonization) [19].

Advances in the management of patients with neurologic disorders and injuries have also resulted in increasing occurrence of infections at these sites, particularly with MRSA [20]. Although microbiology varies depending on type of intervention and antibiotic prophylaxis, more than two thirds of the cases are due to *Staphylococcus* species (approximately half of them *Staphylococcus aureus*), with this percentage increasing over the last two decades [21, 22]. As with bacteremias and intravascular infections, it is imperative to remove foreign devices such as shunts and intraventricular catheters. Treatment should include vancomycin and/or ceftriaxone at doses that will insure adequate penetration into the central nervous system (CNS). Linezolid has also emerged as an alternative agent especially when vancomcyin is not an option due to unachievable trough levels or renal toxicity, due to excellent CNS penetration of linezolid even in the absence of inflamed meninges. Ceftaroline also appears to be an acceptable agent for *Streptococcus pneumoniae* meningitis based on animal data, but human studies are lacking. The following section will focus on the available agents to treat infections caused by GPC in critically ill patients.

## Review

### Linezolid

Linezolid is an oxazolidinone antibiotic that blocks assembly of the initiation complex required for protein synthesis providing broad activity against Gram-positive bacteria with little to no Gram-negative activity [23]. Linezolid has high oral bioavailability (approximately 100%) with toxicity primarily being myelosuppression, peripheral and optic neuropathy, lactic acidosis, and serotonin syndrome [23]. Linezolid is indicated in the US for vancomycin-resistant *Enterococcus faecium* (VRE) infections, including bacteremia; nosocomial pneumonia caused by *Staphylococcus aureus* (MSSA and MRSA), or *Streptococcus pneumoniae* (including multi-drug resistant strains [MDRSP]); complicated and uncomplicated SSTIs; and CAP caused by *Streptococcus pneumoniae* (including MDRSP) and MSSA.

The greatest utility of linezolid seems to be for the treatment of *Staphylococcus aureus* infections, especially nosocomial pneumonia [24–26]. This is especially true for isolates with MICs > 1.0 mg/mL where linezolid appears to be a superior agent [26–28]. Linezolid is also indicated for the treatment of necrotizing pneumonia due to MSSA and MRSA strains secreting the Panton–Valentine leukocidin (PVL) virulence factor given its ability to block toxin production [29] and has been extensively studied for SSTIs, outperforming vancomycin in terms of clinical cures [30–35]. Linezolid has successfully been used off label for the treatment of secondary MRSA bacteremia [36, 37], endocarditis [38, 39], and central nervous system infections [40–42]. The greater efficacy of linezolid over vancomycin observed in some of the above noted clinical studies may be due to the upward drifting MICs of MSSA ansd MRSA to vancomycin as well as the presence of heteroresistance to vancomycin, although not all studies are consistent in demonstrating greater mortality with the presence of heteroresistance [43–50].

Like all other antibiotics, resistance to linezolid has emerged and is a concern given the drug’s potent activity for difficult to treat infections caused by GPC [51]. However, several new oxazolidinone antibiotics are in development, including tedizolid in phase three clinical trials, that offer advantages over linezolid to include coverage of linezolid-resistant isolates and once daily dosing [52, 53].

### Daptomycin

Daptomycin is a bactericidal concentration-dependent lipopeptide that promotes the efflux of potassium out of bacterial cells, leading to cell death. It is indicated for the treatment of SSTIs (6 mg/kg) and *Staphylococcus aureus* bloodstream infections (8 mg/kg) including right-sided infective endocarditis, and it has been used off label for the treatment of difficult central nervous system infections caused by Gram-positive bacteria [52]. Daptomycin should not be used for patients with pneumonia due to the inability to establish non-inferiority to ceftriaxone in a clinical trial, in large part due to the inhibition of daptomycin by surfactant [54, 55]. The main toxicities of daptomycin include eosinophilic pneumonia and skeletal muscle injury.

Guidelines from the Infectious Diseases Society of America (IDSA) for the treatment of MRSA recommend consideration of high-dose (10 mg/kg) daptomycin in patients with persistent MRSA bacteremia associated with vancomycin failure and possibly endocarditis [56]. These recommendations are grounded on the concentration-dependent pharmacokinetic (PK)–pharmacodynamic (PD) profile of daptomycin [57]. Suboptimal daptomycin area under the concentration-time curve (AUC) values indexed to the minimum inhibitory concentration (MIC), or AUC/MIC, have been linked to clinical failure, whereas trough (C_min_) concentrations are correlated with skeletal muscle toxicity [57, 58]. Recently, investigators observed high daptomycin clearance among critically ill patients and significantly lower drug exposures with the use of standard doses [59]. These investigators suggest that daptomycin doses of 750 mg/day may be more effective then the 6 to 8 mg/kg dosing, especially early on when creatinine clearance and volume of distribution may be augmented, especially in septic patients [59].

Several large multicenter observational case series have documented the safety of high-dose daptomycin, to include the treatment of VRE bacteremia which is also an off label indication for its use [60–63]. Moreover, combination with a beta-lactam, trimethoprim/sulfamethoxazole, rifampin or gentamicin have been recommended along with higher dose daptomycin to avoid the emergence of resistance when used as salvage therapy for vancomycin treatment failures [52]. Clinicians should also be aware that recurrent or breakthrough bacteremia following prolonged treatment of *Staphylococcus aureus* or enterococcal infection, to include endocarditis, may signal the emergence of daptomycin resistance, necessitating a change in therapy [11, 64].

### Vancomycin

Vancomycin is a glycopeptides antibiotic with a number of labeled indications for use in the US against GPC, primarily MRSA, to include catheter-related infections, *Clostridium difficile*-associated diarrhea (oral), complicated infections in seriously ill patients, enterocolitis due to *Staphylococcus aureus* (oral), Group B streptococcus (neonatal prophylaxis), meningitis (with third-generation cephalosporin for penicillin-resistant *Streptococcus pneumonia*), pneumonia, prophylaxis against infective endocarditis, and susceptible (MIC ≤1 mcg/mL) Gram-positive infections. There are also many off-label indications where vancomycin is frequently used as first line therapy to include bacteremia, central nervous system infections due to MRSA (brain abscess, subdural empyema, spinal epidural abscess), endocarditis (native valve or prosthetic valve due to *Enterococcus* with vancomycin MIC ≤4 mg/L, streptococci with penicillin MIC >0.5 mg/L or patient intolerance to penicillin, or MRSA), endophthalmitis, SSTIs, prosthetic joint infections, and surgical prophylaxis. The main toxicities of vancomycin for concern in critically ill patients include hypersensitivity reactions, renal toxicity and cytopenias.

The major current problem associated with increasing vancomycin usage over the last several decades is the increasing occurrence of treatment failures due to drug resistance. Rising MICs to vancomycin appears to be the main mechanism associated with these treatment failures [65]. Although uncommon, horizontal transfer of the *vanA* operon from VRE has led to VRSA, while repeated exposure to vancomycin has allowed staphylococci to adapt under selective pressure leading to the emergence of both VISA and heterogeneous-resistant VISA (hVISA) [66, 67]. Surveillance studies have reported the prevalence of hVISA among clinical MRSA isolates to be between zero and 74% [68–73]. The true prevalence of hVISA is difficult to determine since many institutions do not routinely screen for it and there are no standardized methods for rapid detection of hVISA as the ‘gold standard’ population analysis is labor intensive to perform.

Given the emerging resistance of GPC, especially MRSA, to vancomycin, the IDSA has recommended that vancomycin be administered according to body weight (15–20 mg/kg/dose, actual body weight) every 8–12 hours, not to exceed 2 g per dose, in patients with normal renal function (56). However, in seriously ill patients (eg, those with sepsis, meningitis, pneumonia, or infective endocarditis) with suspected MRSA infection, a loading dose of 25–30 mg/kg (actual body weight) may be considered. Vancomycin trough concentrations should be monitored in such patients and maintained between 15–20 μg/mL. Unfortunately, clinical studies do not support an association between greater vancomycin trough levels and improved clinical outcomes supporting the use of alternative agents when suspected or proven infection with high MIC isolates is encountered [26, 33, 74, 75]. Moreover, the MIC test method has a significant impact on vancomycin AUC/MIC estimation [76]. Clinicians should be aware that the current target AUC/MIC of ≥400 for vancomycin was derived using the reference broth microdilution method and does not apply to the use of other automated methods [76].

### Ceftaroline

Ceftaroline is an anti-MRSA cephalosporin that was approved by the FDA in 2010 for the treatment of community-acquired bacterial pneumonia (CABP) and acute bacterial skin and soft structure infections (ABSSSI). Ceftaroline works by binding to penicillin-binding proteins (PBPs) inhibiting their ability to function as transpeptidases in cell wall synthesis. However, it is unique for its affinity for PBP2a and PBP2x providing activity against MRSA and MDRSP including ceftriaxone resistant strains [77]. The approved indications for ceftaroline include SSTIs and CAP at a dose of 600 mg every 12 hours. However, it is important to note that the CAP trials only enrolled patients who were not critically ill [77, 78]. It is not clear whether the approved dose of ceftaroline is adequate for critically ill patients with augmented creatinine clearance and volumes of distribution. In critically ill patients with normal or augmented renal function 600 mg every 8 hours should be considered until more data become available in this population.

Despite ceftaroline having activity against MRSA, little data is available for its use in severe infections caused by Gram-positive bacteria such as infective endocarditis or osteomyelitis. However, a number of case series have recently appeared suggesting that ceftaroline alone, or in combination with another agent, can be used to treat such infections attributed to MRSA or *Enterococcus faecalis*[79–83]. Though limited clinical data supporting ceftaroline for hVISA, VISA or daptomycin non-susceptible *Staphylococcus aureus* infections is currently available, positive *in vitro* data exists to support such off label use [84–86].

### Tigecycline

Tigecycline is a glycylcycline, an analog of tetracyclines with an extended spectrum of activity to include resistant Gram-positive organisms such as MRSA, specific resistant Gram-negative bacteria, to include the extended-spectrum β-lactamase producing *Enterobacteriaceae*, and as salvage therapy for susceptible strains of *Acinetobacter* and other multi-drug resistant (MDR) pathogens. Tigecycline is approved for use by the FDA and European Medicines Agency (EMA) for adults with complicated intra-abdominal infections (cIAIs) and SSTIs as well as for CAP [87–89]. Tigecycline has also been used off label for hospital-acquired pneumonia (HAP) and VAP, diabetic foot infections, urinary tract infections (UTIs), and refractory *Clostridium difficile* infection [90].

A major concern with the use of tigecycline in critically ill patients has to do with the current dosing which is half of the originally planned dosing. This change was made due to perceived unacceptable nausea and emesis at the higher dose. Possibly as a result of this dosing issue several meta-analyses have found the incidence of death to be greater for tigecycline compared to the comparator antibiotics, this was most evident in the nosocomial pneumonia studies [91–93]. However, this mortality excess seems to be driven by infections with Gram-negative bacteria, possible because standard tigecycline doses provide serum concentrations that are below the MICs of most Gram-negative pathogens. Moreover, Ambrose et al. have proposed a tigecycline breakpoint of 0.25 mg/L for *Staphylococcus aureus* and streptococci classifying more isolates as resistant [94]. The use of tigecycline in critically ill patients should be carefully considered in light of the available clinical outcomes data regarding its use.

### Telavancin

Telavancin is a once-daily, intravenous, lipoglycopeptide antibiotic approved in the USA for the treatment of acute bacterial skin and skin structure infections due to Gram-positive pathogens and has recently received approval for the treatment of HAP caused by these pathogens. Unlike other glycopeptides, telavancin maintains its antimicrobial activity against pathogens with decreased susceptibility to glycopeptides, including VISA and hVISA strains, and exhibits more rapid concentration-dependent bactericidal activity against susceptible organisms [95].

In two clinical trials of HAP due to Gram-positive pathogens, particularly MRSA, treatment with telavancin achieved higher cure rates in patients with monomicrobial *Staphylococcus aureus* infection and cure rates comparable to vancomycin in patients with MRSA infection [96]. In patients with mixed Gram-positive/Gram-negative infections, cure rates were higher in the vancomycin group. Incidence and types of adverse events were comparable between the treatment groups. Mortality rates for telavancin-treated versus vancomycin-treated patients were 21.5% versus 16.6% and 18.5% versus 20.6% for the two trials. Increases in serum creatinine level were more common in the telavancin group (16% vs 10%) [96].

Due to updated FDA guidance [97] for future antibiotic clinical trials of bacterial nosocomial pneumonia that recommend using diagnostic criteria from the American Thoracic Society/Infectious Diseases Society of America (ATS/IDSA) guidelines [98], and using a primary end point of 28-day all-cause mortality, a *post-hoc* reanalysis of the two HAP studies was undertaken [99]. Clinical cure rates at final follow-up were determined in the refined all-treated (AT) and clinically-evaluable (CE) groups (ATS/IDSA-AT and ATS/IDSA-CE, respectively) and the exploratory end point of 28-day survival was evaluated in the ATS/IDSA-AT group. Non-inferiority of telavancin versus vancomycin was demonstrated, with similar cure rates in the ATS/IDSA-AT (59% versus 59%, respectively) and ATS/IDSA-CE groups (83% versus 80%, respectively). Cure rates favored telavancin in ATS/IDSA-CE patients where *Staphylococcus aureus* was the sole pathogen (86% versus 75%). Overall, 28-day survival was similar in the telavancin (76%) and vancomycin (77%) groups, but lower in telavancin-treated patients with pre-existing moderate-to-severe renal impairment (CL_CR_ <50 ml/min). The FDA approval indicates that telavancin should only be administered to patients with moderate-to-severe renal impairment if treatment benefit outweighs risk, or if no suitable alternatives are available.

## Conclusions

The rise in infections attributed to GPC in critically ill patient mandates that clinicians treating these individuals be familiar with the pathogen types, virulence factors, and susceptibilities of GPC in their local practice areas. Moreover, the availability of MICs, especially for vancomycin and daptomycin in MRSA, should help direct the use of these agents, as well as the new antimicrobials targeting GPC. This is especially important in potentially life-threatening infections or infections associated with foreign bodies. Moreover, there is a need for the development of non-traditional agents such as vaccines and monoclonal antibodies directed against GPC such as MRSA in order to help prevent these infections and improve their outcomes [100].

## Authors’ information

MHK holds the Virginia E. and Sam J. Golman Chair in Respiratory Intensive Care Medicine and is full professor At Washington University.

## Abbreviations

ABSSSI: Acute bacterial skin and soft structure infections
AT: All-treated
ATSD: American Thoracic Society
AUC: Area under the curve
CABP: Community-acquired bacterial pneumonia
CAP: Community-acquired pneumonia
CE: Clinically-evaluable
cIAI: Complicated intra-abdominal infection
CLCR: Creatinine clearance
Cmin: Concentration minimum
CNS: Central nervous system
GPC: Gram-positive cocci
HAP: Hospital-associated pneumonia
HCAP: Healthcare-associated pneumonia
hVISA: Heteroresistant vancomycin-intermediate *Staphylococcus aureus*
ICU: Intensive care unit
IDSA: Infectious Disease Society of America
MDRSP: Multidrug-resistant *Streptococcus pneumoniae*
MIC: Minimum inhibitory concentration
MSSA: Meticillin-susceptible *Staphylococcus aureus*
MRSA: Methicillin-resistant *Staphylococcus aureus*
PBP: Penicillin binding protein
PD: Pharmacodynamic
PK: Pharmacokinetic
PVL: Panton–Valentine leukocidin
SSTI: Skin and soft tissue infections
UTI: Urinary tract infection
VAP: Ventilator-associated pneumonia
VISA: Vancomycin intermediate *Staphylococcus aureus*
VRSA: Vancomycin-resistant *Staphylococcus aureus*
VRE: Vancomycin-resistant enterococci.
